# InterPro: the protein sequence classification resource in 2025

**DOI:** 10.1093/nar/gkae1082

**Published:** 2024-11-20

**Authors:** Matthias Blum, Antonina Andreeva, Laise Cavalcanti Florentino, Sara Rocio Chuguransky, Tiago Grego, Emma Hobbs, Beatriz Lazaro Pinto, Ailsa Orr, Typhaine Paysan-Lafosse, Irina Ponamareva, Gustavo A Salazar, Nicola Bordin, Peer Bork, Alan Bridge, Lucy Colwell, Julian Gough, Daniel H Haft, Ivica Letunic, Felipe Llinares-López, Aron Marchler-Bauer, Laetitia Meng-Papaxanthos, Huaiyu Mi, Darren A Natale, Christine A Orengo, Arun P Pandurangan, Damiano Piovesan, Catherine Rivoire, Christian J A Sigrist, Narmada Thanki, Françoise Thibaud-Nissen, Paul D Thomas, Silvio C E Tosatto, Cathy H Wu, Alex Bateman

**Affiliations:** European Molecular Biology Laboratory, European Bioinformatics Institute (EMBL-EBI), Wellcome Genome Campus, Hinxton CB10 1SD, UK; European Molecular Biology Laboratory, European Bioinformatics Institute (EMBL-EBI), Wellcome Genome Campus, Hinxton CB10 1SD, UK; European Molecular Biology Laboratory, European Bioinformatics Institute (EMBL-EBI), Wellcome Genome Campus, Hinxton CB10 1SD, UK; European Molecular Biology Laboratory, European Bioinformatics Institute (EMBL-EBI), Wellcome Genome Campus, Hinxton CB10 1SD, UK; European Molecular Biology Laboratory, European Bioinformatics Institute (EMBL-EBI), Wellcome Genome Campus, Hinxton CB10 1SD, UK; European Molecular Biology Laboratory, European Bioinformatics Institute (EMBL-EBI), Wellcome Genome Campus, Hinxton CB10 1SD, UK; European Molecular Biology Laboratory, European Bioinformatics Institute (EMBL-EBI), Wellcome Genome Campus, Hinxton CB10 1SD, UK; European Molecular Biology Laboratory, European Bioinformatics Institute (EMBL-EBI), Wellcome Genome Campus, Hinxton CB10 1SD, UK; European Molecular Biology Laboratory, European Bioinformatics Institute (EMBL-EBI), Wellcome Genome Campus, Hinxton CB10 1SD, UK; European Molecular Biology Laboratory, European Bioinformatics Institute (EMBL-EBI), Wellcome Genome Campus, Hinxton CB10 1SD, UK; European Molecular Biology Laboratory, European Bioinformatics Institute (EMBL-EBI), Wellcome Genome Campus, Hinxton CB10 1SD, UK; Department of Structural and Molecular Biology, University College London, Gower St, Bloomsbury, London WC1E 6BT, UK; European Molecular Biology Laboratory, Structural and Computational Biology Unit, Meyerhofstraße 1, 69117 Heidelberg, Germany; Swiss-Prot Group, Swiss Institute of Bioinformatics, CMU, 1 rue Michel Servet, CH-1211, Geneva, Switzerland; Google DeepMind, Cambridge, MA 02142, USA; Medical Research Council Laboratory of Molecular Biology, Cambridge Biomedical Campus, Francis Crick Ave, Trumpington, Cambridge CB2 0QH, UK; National Center for Biotechnology Information, National Library of Medicine, National Institutes of Health, 8600 Rockville Pike, Bethesda, MD 20894, USA; Biobyte Solutions GmbH, Bothestr 142, 69126 Heidelberg, Germany; Google DeepMind, 75009 Paris, France; National Center for Biotechnology Information, National Library of Medicine, National Institutes of Health, 8600 Rockville Pike, Bethesda, MD 20894, USA; Google DeepMind, 8002 Zürich, Switzerland; Division of Bioinformatics, Department of Population and Public Health Sciences, University of Southern California, Los Angeles, CA 90033, USA; Protein Information Resource, Georgetown University Medical Center, WA, DC 20007, USA; Department of Structural and Molecular Biology, University College London, Gower St, Bloomsbury, London WC1E 6BT, UK; Medical Research Council Laboratory of Molecular Biology, Cambridge Biomedical Campus, Francis Crick Ave, Trumpington, Cambridge CB2 0QH, UK; Department of Biomedical Sciences, University of Padova, Padova 35121, Italy; Swiss-Prot Group, Swiss Institute of Bioinformatics, CMU, 1 rue Michel Servet, CH-1211, Geneva, Switzerland; Swiss-Prot Group, Swiss Institute of Bioinformatics, CMU, 1 rue Michel Servet, CH-1211, Geneva, Switzerland; National Center for Biotechnology Information, National Library of Medicine, National Institutes of Health, 8600 Rockville Pike, Bethesda, MD 20894, USA; National Center for Biotechnology Information, National Library of Medicine, National Institutes of Health, 8600 Rockville Pike, Bethesda, MD 20894, USA; Division of Bioinformatics, Department of Population and Public Health Sciences, University of Southern California, Los Angeles, CA 90033, USA; Department of Biomedical Sciences, University of Padova, Padova 35121, Italy; Institute of Biomembranes, Bioenergetics and Molecular Biotechnologies, National Research Council (CNR-IBIOM), Bari 70126, Italy; Protein Information Resource, Georgetown University Medical Center, WA, DC 20007, USA; European Molecular Biology Laboratory, European Bioinformatics Institute (EMBL-EBI), Wellcome Genome Campus, Hinxton CB10 1SD, UK

## Abstract

InterPro (https://www.ebi.ac.uk/interpro) is a freely accessible resource for the classification of protein sequences into families. It integrates predictive models, known as signatures, from multiple member databases to classify sequences into families and predict the presence of domains and significant sites. The InterPro database provides annotations for over 200 million sequences, ensuring extensive coverage of UniProtKB, the standard repository of protein sequences, and includes mappings to several other major resources, such as Gene Ontology (GO), Protein Data Bank in Europe (PDBe) and the AlphaFold Protein Structure Database. In this publication, we report on the status of InterPro (version 101.0), detailing new developments in the database, associated web interface and software. Notable updates include the increased integration of structures predicted by AlphaFold and the enhanced description of protein families using artificial intelligence. Over the past two years, more than 5000 new InterPro entries have been created. The InterPro website now offers access to 85 000 protein families and domains from its member databases and serves as a long-term archive for retired databases. InterPro data, software and tools are freely available.

## Graphical Abstract

**Graphical Abstract:**
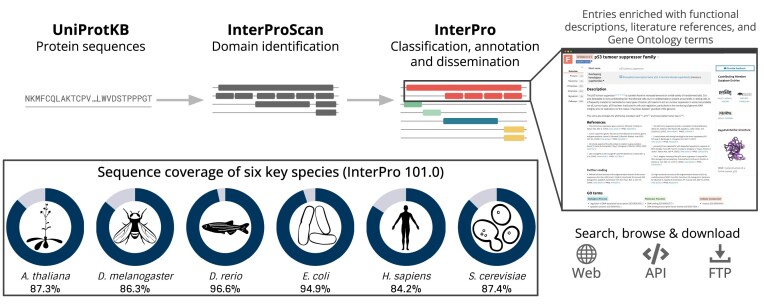

## Introduction

The rapid advancements in genomic technologies, coupled with substantial decreases in the cost of sequencing, have brought forth an era where large-scale sequencing projects generate data at an unprecedented rate. However, the surge in data volume has outpaced our capabilities to characterise sequences through experimental methods alone. Consequently, there is a strong need for sophisticated computational tools and databases to manage, annotate and interpret these vast repositories of biological information.

Launched in 1999, InterPro aims to meet this challenge by integrating diverse information about protein families, domains and functional sites from specialist member databases into a single cohesive resource. These databases include CATH-Gene3D (1), the Conserved Domains Database (CDD) (2), HAMAP (3), NCBIFAM (4), PANTHER (5), PIRSF (6), Pfam (7), PRINTS (8), PROSITE (9), the Structure-Function Linkage Database (SFLD) (10), SMART (11) and SUPERFAMILY (12). They utilise predictive models – such as profile hidden Markov models (HMM), position-specific scoring matrices, and regular expressions, collectively referred to as *signatures* – to search protein sequences and assign potential functions.

InterPro consolidates and cross-references these diverse annotations, producing a comprehensive overview of protein families, domains and functional sites. Each InterPro entry is assigned a unique accession number, name and short name and is annotated with a descriptive abstract and Gene Ontology (GO) terms (13) that can be assigned to all sequences matched by that entry (14). This integrative approach not only reduces redundancy but also enhances the robustness of protein annotations available to the scientific community.

InterPro also provides comprehensive information about sequence features, including consensus annotations of long-range intrinsic disorder regions predicted by MobiDB-lite (15), and predictions of signal peptides, transmembrane regions and coiled-coils using the SignalP (16), Phobius (17), TMHMM (18) and Coils (19) software packages. Additionally, InterPro offers functional classification of protein domains through FunFam (20), facilitating the identification of evolutionary relationships and functional properties. Furthermore, InterPro includes annotations from AntiFam (21), a tool designed to detect spurious open reading frames (ORFs).

InterPro and its associated software are extensively disseminated and utilised by the scientific community, such that it is recognised as a Core Data Resource by ELIXIR (22) and as a Global Core Biodata Resource by the Global Biodata Coalition (23). Over 40 million sequence searches were processed between January 2023 and June 2024 by the InterProScan web service, which facilitates the analysis of user-submitted sequences via the EMBL-EBI Job Dispatcher (24).

A primary application of InterPro data is its integration into UniProtKB (25), where InterPro annotations underpin the automatic annotation of proteins (26). To support this task, InterPro matches are computed weekly using the InterProScan software package (27) against new sequences deposited in the UniProt Archive (UniParc), a comprehensive and non-redundant database containing the majority of publicly available protein sequences. This process ensures that new sequences are promptly and accurately annotated. Additionally, InterPro data are integral to various annotation pipelines, including Ensembl (28), Ensembl Genomes (29), PDBe (30), Blast2GO (31), Genome Properties (32), MGnify (33) and MGnify Genomes (34).

InterPro's release cycle aligns with UniProt, making new releases of InterPro publicly available every 8 weeks, 1 day after each UniProt release.

### Data update

Member database signatures are not automatically incorporated into InterPro; instead, they undergo a thorough manual inspection and integration process. Matches between these signatures and the most recent version of UniProtKB are evaluated to eliminate any false positives. Signatures corresponding to the same biological entity are consolidated into unified InterPro entries, thereby reducing redundancy. For instance, signatures from CDD, PROSITE Profiles, Pfam and SMART, which represent the CUB domain (cd00041, PS01180, PF00431, SM00042, respectively), are integrated into a single InterPro entry (IPR000859).

New InterPro entries are subject to manual annotation, including assignment of a name, short name, descriptive abstract and GO terms that are applied consistently to all matched proteins. Hierarchical relationships are established among evolutionarily related InterPro entries, linking smaller, functionally specific subfamilies of larger families, or subclasses of broader classes of domains. Bi-monthly reviews are conducted on annotation and sequence match information following UniProtKB updates, with InterPro entries adjusted based on revised sequence information or updated biological knowledge (e.g. newly characterised protein functions). Despite requiring significant curation effort, these updates are crucial for maintaining annotation accuracy, especially given the dynamic nature of the underlying data.

With each release of InterPro, one or more of its constituent member databases may undergo updates, resulting in the continuous addition of new signatures and entries into the resource. Since the last update publication (35), there have been 11 public releases of InterPro, during which 8734 new signatures have been integrated into 7963 new InterPro entries. The most recent release, version 101.0, includes 84 588 member database signatures, 69% of which are integrated into 45 899 InterPro entries. The percentage of signatures integrated into InterPro for each member database is shown in Table 1.

**Table 1.** Release version and number of member database signatures integrated into InterPro version 101.0

Member database	Release number	Total signatures	Integrated signatures
CATH-Gene3D	4.3.0	6631	2822 (42.6%)
CDD	3.20	18 882	4908 (26.0%)
HAMAP	2023_05	2389	2384 (99.8%)
NCBIFAM	15.0	7289	5884 (80.7%)
PANTHER	18.0	15 693	10 520 (67.0%)
Pfam	37.0	21 979	21 171 (96.3%)
PIRSF	3.10	3285	3230 (98.3%)
PRINTS	42.0	2106	1939 (92.1%)
PROSITE Patterns	2023_05	1311	1283 (97.9%)
PROSITE Profiles	2023_05	1379	1,21 (93.6%)
SFLD	4	303	159 (52.5%)
SMART	9.0	1322	1274 (96.4%)
SUPERFAMILY	1.75	2019	1643 (81.4%)

In UniProtKB, 81.8% of the sequences have at least one match to an InterPro entry (see Table 2). Although sequence coverage has decreased by 0.2% over the past 2 years, this decline should be considered in the context of substantial growth within UniProtKB during the same period, with InterPro annotations now covering an additional 18 million sequences. Meanwhile, the number of sequences in UniParc has increased by 56%, and InterPro coverage of UniParc has grown by 1.1%.

**Table 2.** Coverage of UniProtKB and UniParc (non-redundant archive of protein sequences) by InterPro entries (version 101.0)

Sequence database	Number of sequences	Number of sequences with one or more matches to InterPro
UniProtKB/reviewed	571 864	553 706 (96.8%)
UniProtKB/unreviewed	245 324 902	200 675 244 (81.8%)
UniProtKB (total)	245 896 766	201 228 950 (81.8%)
UniParc	808 031 558	654 323 531 (81.0%)

**Figure 1.**
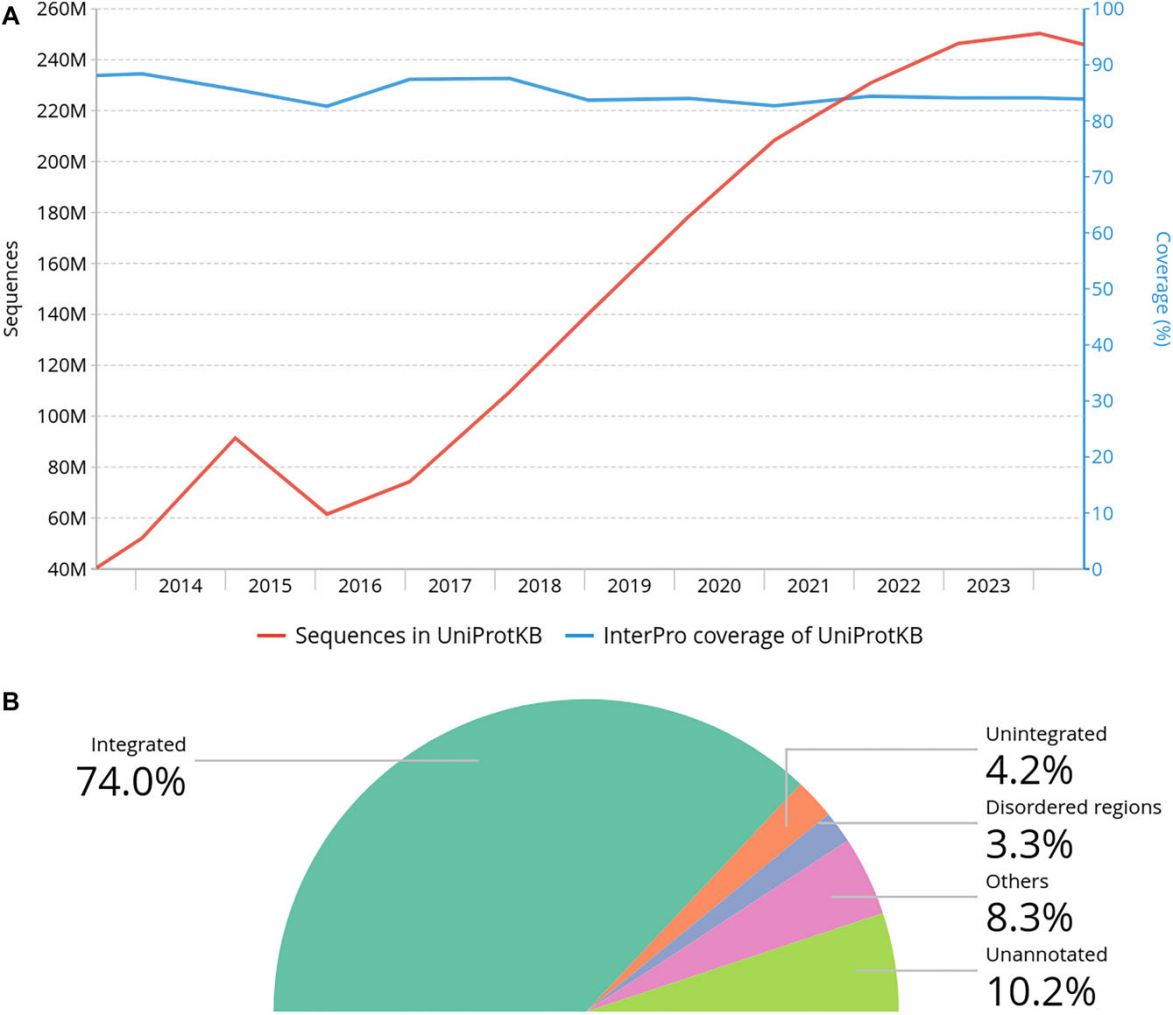
InterPro coverage of UniProtKB. (**A**) InterPro coverage of UniProtKB sequences alongside the growth of UniProtKB over time (January 2014–July 2024). (**B**) InterPro coverage of amino acid residues in UniProtKB, categorised as follows: residues covered by signatures already integrated into InterPro, signatures from member databases that are awaiting integration, intrinsically disordered regions and regions predicted to be signal peptides, transmembrane domains, or coiled-coils. The remaining residues are classified as unannotated.

As UniProtKB continues to expand, maintaining or improving InterPro coverage is increasingly challenging. Larger, well-characterised protein families have already been integrated, and newer entries often focus on more narrowly defined taxonomic groups. Despite a 371% increase in the number of sequences in UniProtKB over the past decade, InterPro has managed to sustain a sequence coverage above 80% (see Figure 1A). This highlights ongoing efforts to enhance annotation across a rapidly growing sequence database.

Beyond sequence coverage, we have also evaluated the amino acid residue coverage of InterPro and its member databases. Overall, 89.8% of residues in UniProtKB receive some level of annotation. Figure 1B illustrates the cumulative unique residue coverage: (i) InterPro entries cover 64.4 billion out of 87 billion residues (74%); (ii) signatures from member databases pending integration into InterPro cover 4.2%; (iii) residues found in intrinsically disordered regions account for 3.3%; (iv) residues associated with other sequence features, such as coiled-coil regions, transmembrane regions and signal peptides, cover 8.3%; and (v) residues with no annotation make up the remaining portion.

### New developments

#### InterPro: the new home of Pfam data

As reported in our previous publication, we decided to decommission the Pfam website (formerly accessible at http://pfam.xfam.org) due to challenges in maintaining and updating its legacy infrastructure. In August 2022, we posted a notice on the Pfam website, informing users of an impending automatic redirection to the InterPro website starting in October 2022. With the initiation of this redirection in October 2022, we provided access to a legacy version of the Pfam website at http://pfam-legacy.xfam.org for users who had not yet migrated.

Although the legacy site was initially scheduled for shutdown in January 2023, it remained available until Summer 2023 to facilitate user transition. This transition was supported by several blog posts, X (formerly Twitter) updates and webinars to ensure a smooth migration of the Pfam userbase to InterPro. The former Pfam website (http://pfam.xfam.org) now hosts a static page that replicates the appearance of the old site, with its features (search, browse, etc.) redirecting to the corresponding pages on the InterPro website.

Pfam is now exclusively accessible through InterPro, where all critical features from the original Pfam site are available. Users can also access Pfam data programmatically via the InterPro REST API and annotate sequences with Pfam through InterProScan, which is available both as a web service and a downloadable standalone program. By moving away from a legacy codebase with increasing maintenance demands, Pfam now focuses more on its core mission of building protein families. This shift allows for more frequent updates, which will be seamlessly integrated into InterPro releases. All past Pfam release files remain accessible via the EMBL-EBI FTP site, with future release files to be made available there as well.

### Leveraging AI to describe protein families

InterPro entries are derived from member database signatures, with annotations for new entries often sourced from these databases. When member database signatures lack annotations, Interpro curators must create descriptions *de novo*, frequently using scientific literature. This process is laborious and slows down the integration of signatures. This issue is particularly notable for PANTHER, a vast collection of protein families. PANTHER ranks as the third largest member database by the number of signatures it provides (preceded by CDD and Pfam) and as the second largest by the number of UniProtKB sequences matched (after Pfam). However, no PANTHER signatures are accompanied by descriptive abstracts, resulting in a lower integration rate. Moreover, the rising publication rate, combined with a limited number of InterPro curators, exacerbates the challenge.

Recent advancements in large language models (LLMs) have reached a quality level sufficient for application in some curation tasks. LLMs, a class of machine learning models featuring billions of parameters, excel at predicting subsequent tokens in a sequence. A notable application of LLMs is LitSumm (36), a method that generates automated summaries for non-coding RNA (ncRNA) genes. LitSumm extracts and selects relevant sentences from the literature using ncRNA identifiers and names, instructs a LLM to generate a summary, and then further directs the LLM to check and refine the summary for consistency. However, this method is less effective with PANTHER signatures since their accession numbers are infrequently cited in publications and their names are often non-descriptive (e.g. Uncharacterised, Expressed protein, Hypothetical protein). Nonetheless, 70% of unintegrated PANTHER signatures match at least one sequence from Swiss-Prot, the manually curated section of UniProtKB. To address the lack of descriptions, we developed an alternative approach using GPT-4 models from OpenAI (37). For PANTHER signatures with Swiss-Prot matches, we extracted relevant information from Swiss-Prot entries, including protein names and general annotations (function, activity regulation, similarity). We then instructed the model to summarise the overall function of the family using this information and the knowledge embedded in the model. Manual assessments of a subset of these summaries showed high-quality ratings for the majority of them. Consequently, we released 5491 descriptions of PANTHER families in InterPro 97.0. We further refined our method to include the generation of names and short names for each family, facilitating the automatic creation of InterPro entries.

**Figure 2.**
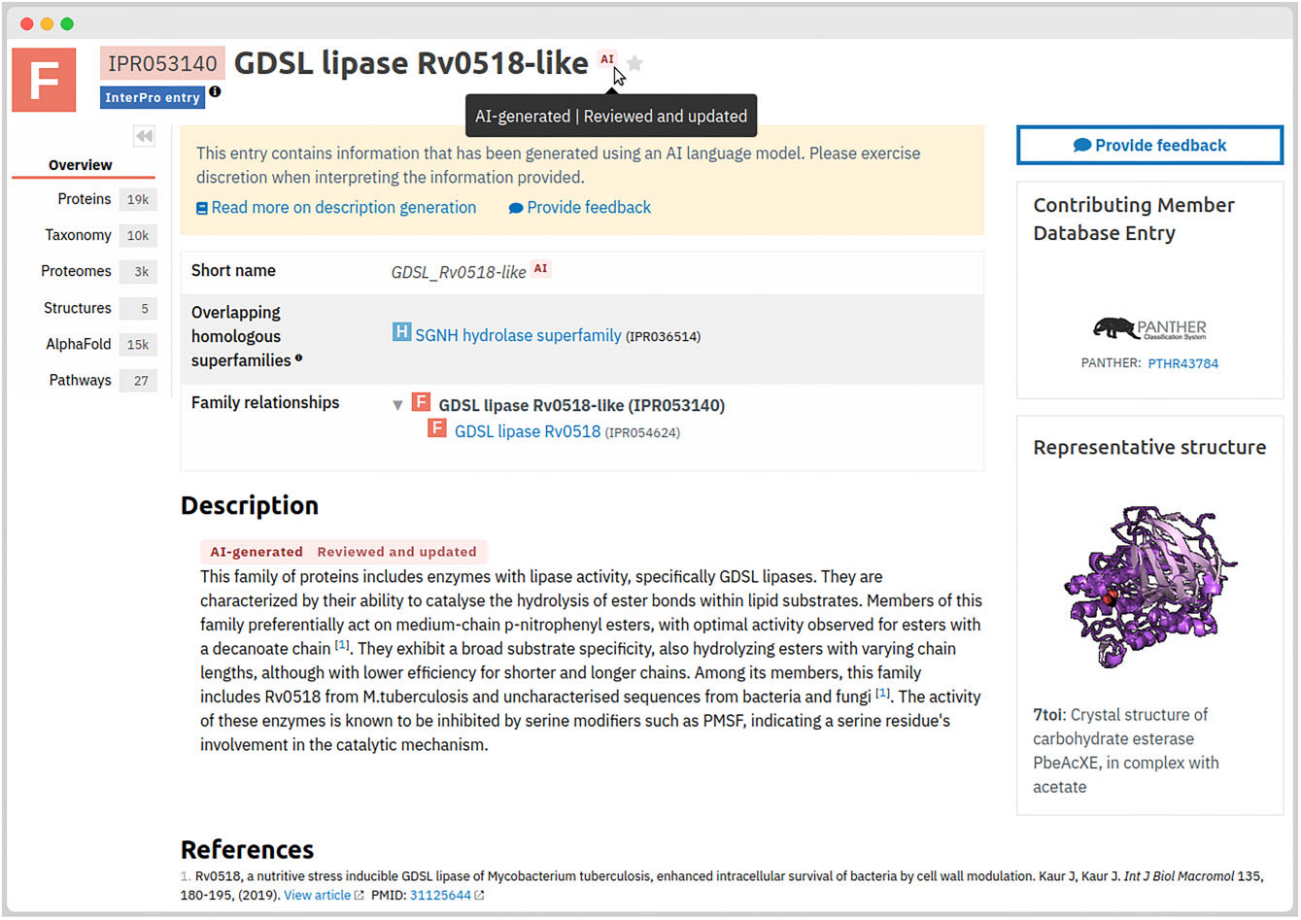
Example of InterPro entry IPR053140, automatically generated using AI annotations for PANTHER family PTHR43784. The entry features an ‘AI’ label next to the entry name, short name and description to indicate its AI-generated origin. In this instance, a curator has reviewed, updated the description, and included a reference to a relevant scientific publication for supporting evidence. Users are encouraged to use the ‘Provide feedback’ button to report any inaccuracies or suggest improvements.

Between InterPro 99.0 and 101.0, we automatically generated 3887 InterPro entries based on AI-generated annotations for 3316 PANTHER families, 507 NCBIFAM families, and 64 CATH-Gene3D superfamilies. These entries are clearly marked as AI-generated and unreviewed on the InterPro website (see Figure 2). Each page containing AI-generated annotations includes a warning notice with a link to the InterPro documentation, explaining how the annotations were generated, which model was used, and providing an example input context. This documentation will be updated as needed if we change the annotation method or model. Additionally, we updated our internal curation application to allow curators to review and approve these annotations, with the reviewed status reflected in subsequent releases.

Employing smaller local models is one of the possibilities of speeding up and decreasing the cost of using LLMs. We explored this possibility with fine-tuning a SciPhi-Mistral-7B-32k model using PANTHER families, by learning to imitate GPT-4 responses. This particular model was pretrained from Mistral 7B v0.1 (38) on scientific texts, which makes it suitable for describing protein families.

To fine-tune the SciPhi-Mistral model, we used a collection of 5559 PANTHER families that we had previously described using GPT-4. For each family, we provided a prompt containing the information from Swiss-Prot proteins and specific instructions for GPT-4, along with the corresponding response generated by GPT-4 based on the prompt. The only change in the prompts was additional markdown for the SciPhi-Mistral model. The markdown contained tags [SYS], [CONT], [INST], [ANS] (for ‘system’, ‘context’, ‘instruction’ and ‘answer’, respectively) and corresponding closing tags helping the model to parse the input. The dataset was divided into training (5473 families) and testing (86 families) subsets. We ran the fine-tuning for one epoch, where the SciPhi-Mistral model was learning to generate responses similar to GPT-4 responses.

To run the evaluation, we randomly selected 56 families from the testing subset and generated three types of responses for each: (i) a response generated by GPT-4; (ii) a response generated by the baseline, non-fine-tuned SciPhi-Mistral-7B-32k model; (iii) a response generated by our fine-tuned SciPhi-Mistral-7B-32k model.

**Figure 3.**
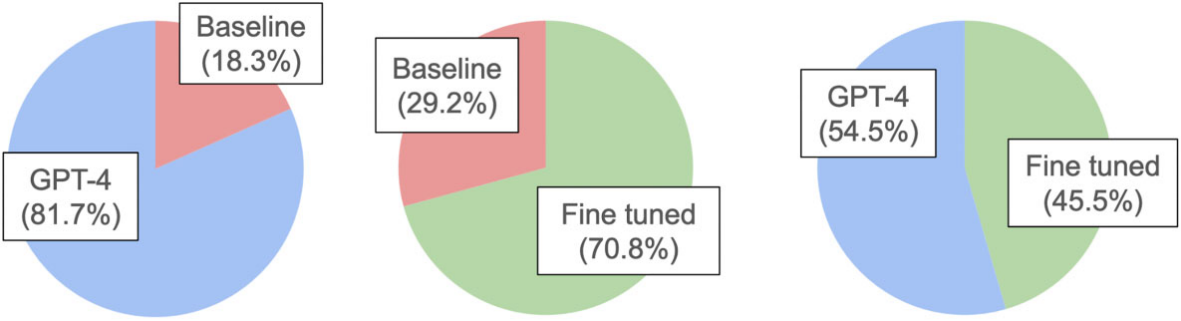
Percentages of pairs where one or another model response was preferred by the InterPro curators.

To compare the models, we created 168 response pairs by considering all possible pairwise comparisons for each of the 56 families. These pairs were loaded into Label Studio, where a labelling task was created for each of the 5 human InterPro curators. For each pair, we asked the curators to decide which of the two models’ responses they prefer. No other information except for the textual descriptions was given. The evaluation was performed independently by each curator. After this, their responses were aggregated as votes with the same weight. The results of the evaluation are presented in Figure 3. The baseline model predictions are outperformed by both GPT-4 and the fine tuned model by a large margin – GPT-4 was preferred to the baseline SciPhi-Mistral in 81.7% pairs and fine tuned SciPhi-Mistral was preferred to the baseline model in 70.8% pairs. However, responses generated by the fine tuned model were preferred by the curators only slightly less than the ones by GPT-4 model (45.5 versus 54.5%). These results show that the smaller local SciPhi-Mistral-7B-32k can be successfully employed for the description generation task, with the results comparable in quality to the GPT-4 model.

Despite this success, the SciPhi-Mistral-generated annotations are not currently used on the InterPro website. Although SciPhi-Mistral is a cost-effective alternative to GPT-4, the relatively small input context for many families, due to the limited number of Swiss-Prot matches, makes GPT-4′s usage manageable in terms of cost.

We plan to continue evaluating both commercial and non-commercial models. As part of this ongoing work, we will always clearly indicate the model used to generate annotations and provide details on the instructions given.

### Website and API improvements

#### Enhancing the integration and visualisation of AlphaFold predicted structures in InterPro

Over 85% of UniProtKB entries now feature predicted protein structures generated by AlphaFold, an artificial intelligence method developed by Google DeepMind that infers protein structures from their amino acid sequences (39,40). In our previous publication, we reported the integration of the Mol* 3D molecular viewer (41) to enable users to visualise predicted structures, which are colour-coded by the predicted local distance difference test (pLDDT), a per-residue confidence measure. The pLDDT score ranges from 0 to 100, with higher scores indicating greater confidence. We have now enhanced our protein viewer to also display the pLDDT confidence scores as a 2D track.

Initially, there were two access points for AlphaFold models. The first is via the ‘AlphaFold’ tab on the page of a protein with a predicted structure. The second is through the ‘AlphaFold’ tab on InterPro entry pages, where an example AlphaFold model is shown along with a table listing other available models for that entry. We have now extended the second access point to include all member database signatures, allowing users to access the predicted structures of sequences matched by any member database signature, regardless of whether they have been integrated into an InterPro entry.

Previously, protein structures for Pfam families were predicted using RoseTTAFold (42) and were available for both Pfam families and the InterPro entries in which they were integrated. RoseTTAFold models provided a single, combined view for the family, but if Pfam domain boundaries were incorrect, the structural model would be incomplete. With the majority of Pfam families now having hundreds or thousands of AlphaFold predictions, we have decided to retire the RoseTTAFold models.

Because the sequence associated with a UniProtKB entry may be updated over time, there are instances where an AlphaFold structure was predicted using an earlier version of the sequence. When sequence mismatches are detected, we display a warning message to inform users that the prediction may not accurately represent the current protein structure due to this discrepancy.

With the availability of AlphaFold models for the majority of sequences in UniProtKB, users might be primarily interested in proteins with a predicted structure. Therefore, we have updated all pages listing proteins to indicate whether a protein has a predicted AlphaFold model. When this is the case, we display a link to the page where users can visualise the 3D molecular structure. For example, proteins matched by InterPro can be viewed at https://www.ebi.ac.uk/interpro/protein/UniProt/entry/InterPro/, proteins matched by the Pfam SH2 domain at https://www.ebi.ac.uk/interpro/entry/pfam/PF00017/protein/UniProt/, and human proteins at https://www.ebi.ac.uk/interpro/taxonomy/uniprot/9606/protein/UniProt/.

Moreover, enabling structural searches has become a critical feature for researchers. To enhance this capability, we have integrated direct links to the Foldseek webserver (43) on protein pages with AlphaFold predictions and PDB structure pages within InterPro. This one-click access to Foldseek automatically uploads the relevant PDB file to the server making user interactions smoother and faster. By leveraging the extensive structural data now available, this addition significantly improves the use of InterPro for researchers investigating protein function and relationships through structural comparisons.

### Improving the visualisation of protein domains and features

Effective visualisation is essential for presenting large volumes of information in a compact, easily interpretable format. InterPro facilitates the visualisation of protein sequence annotations from all its member databases, as well as other regions of interest, such as signal peptides, transmembrane domains, coiled-coils and disordered regions. Although the breadth and diversity of annotations make InterPro a comprehensive resource for protein sequence analysis, they can also lead to potential confusion, especially on protein pages that display over 30 annotation tracks.

**Figure 4.**
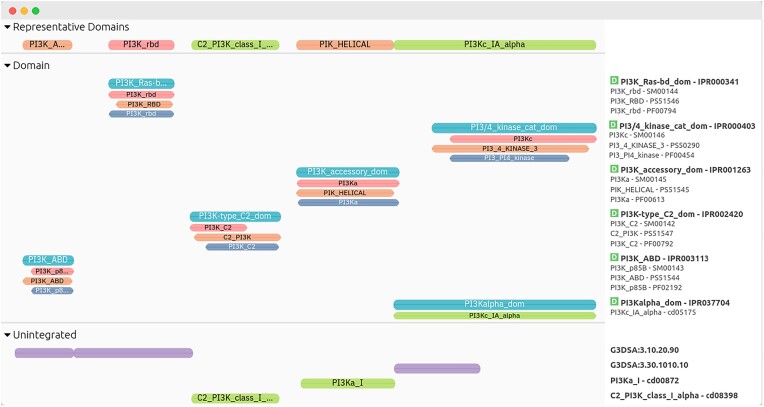
InterPro annotations for the human PIK3CA protein (UniProtKB accession Q15648). The ‘Representative Domains’ track displays selected domains from InterPro member databases, chosen to maximise coverage and minimise overlap, providing users with a comprehensive overview of the sequence's domain organisation. The ‘Domain’ section includes InterPro entries classified as domains, along with the underlying member database signatures that match the protein sequence. The ‘Unintegrated’ section lists annotations from member database signatures that have not yet been integrated into an InterPro entry.

Previously, the Pfam website presented the organisation of domains for a given UniProt entry on a single line. However, Pfam domains do not overlap, and since InterPro integrates domains from multiple member databases, displaying them on a single line would result in overlaps. To enhance clarity and conciseness in visualising protein domains, we now display representative domains at the top of all other tracks in our protein viewer (see Figure 4).

Representative domains are selected through an automated process that compares domains from Pfam, CDD, PROSITE Profiles, SMART and NCBIFAM. The smallest set of domains is chosen to maximise sequence coverage while avoiding overlaps. These representative domains are accessible via the InterPro website, the InterPro REST API and can be downloaded from our FTP in the ‘match_complete.xml’ file, which includes all matches from member database signatures found in UniProtKB.

**Figure 5.**
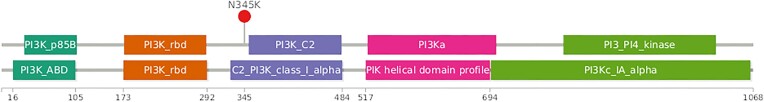
N345K variant in the context of the human PIK3CA protein (UniProtKB accession P42336). The top track illustrates the organisation of Pfam domains, while the bottom track highlights representative domains, with the CDD C2 domain selected over the Pfam C2 domain. Although the N345K mutation, known to reside within the C2 domain, appears outside the Pfam C2 domain, it is located within the boundaries of the CDD C2 domain.

Using representative domains selected from several databases offers a more reliable approach to domain annotation. This strategy helps to mitigate and rectify occasional discrepancies that can arise when relying on a single source. For instance, in the case of the human PIK3CA protein, the N345K variant has been reported to lie within the C2 domain (44). However, if one were to rely solely on Pfam domain annotations, the N345K mutation would incorrectly appear outside the C2 domain due to the shorter boundaries of the Pfam C2 domain. By using representative domains, the CDD C2 domain is selected instead, accurately positioning the N345K variant within the C2 domain (see Figure 5). This example highlights the importance of integrating multiple domain databases to ensure accurate variant classification and reduce the risk of misinterpretation.

Since our last publication, we have significantly enhanced the InterPro protein viewer by integrating annotations from several external resources, broadening the scope and depth of the data presented. Among these additions are short linear motifs (SLiMs) sourced from the Eukaryotic Linear Motif (ELM) database (45), a manually curated collection of motif instances derived from experimental literature. SLiMs play crucial roles in mediating protein-protein interactions and other regulatory processes, and their inclusion expands InterPro to include motifs that are often difficult to represent with HMMs.

We have also incorporated data on intrinsically disordered regions from DisProt (46), a comprehensive database that curates experimental evidence of protein disorder from scientific literature. Intrinsically disordered regions are essential for understanding protein flexibility, conformational changes and interactions that do not fit traditional structure-function paradigms.

Additionally, we now display protein repeats retrieved from RepeatsDB (47), a specialised database that annotates tandem repeat structures in proteins. These repeats are often associated with specific functional or structural properties, and their visualisation within the context of InterPro annotations allows for a more detailed analysis of protein architecture.

Disease mutations and variants are integrated into the protein viewer from the Proteins API (48). This integration enables biomedical researchers to correlate protein function and conservation with natural variations and disease-causing mutations. By placing these variants within the broader context of InterPro's extensive protein annotations, researchers can more effectively explore the potential impact of genetic variations on protein function and disease mechanisms.

Lastly, we updated the protein viewer to use the latest version of Nightingale, a library of bioinformatics web components (49). By delegating the visualisation logic to Nightingale, which adheres to strict web standards, we are making the InterPro codebase easier to maintain. Furthermore, the latest version of Nightingale is written in TypeScript, a superset of JavaScript that introduces static types. This enhancement improves code quality and developer experience by providing better tooling for catching errors early and facilitating more robust and readable code.

### Enhancing InterProScan's web-based sequence analysis

One of the key features of the InterPro website is the ability for researchers to scan their sequences using InterProScan directly within their web browser. This functionality is particularly beneficial for researchers who may lack access to substantial computing power or the technical expertise required to install and operate command-line tools like InterProScan. It enables users to annotate their sequences seamlessly online. Previously, users were limited to submitting only one protein sequence per search.

The InterProScan search functionality on the InterPro website is powered by the EMBL-EBI Job Dispatcher sequence analysis tools framework. Through collaboration with the team responsible for this framework, we have enhanced the system to allow users to submit up to 100 nucleotide or protein sequences per run. Additionally, we have redesigned the sequence submission, run monitoring, and results pages to streamline the browsing and analysis of results for multiple sequences.

### Increasing the integration and accessibility of AntiFam

In our previous publication, we reported the integration of AntiFam into InterProScan, enabling researchers to detect spurious ORFs, a feature particularly useful for quality control in metagenomic and genomic studies. AntiFam hits identified in UniProtKB were displayed alongside other domains and regions of interest within the protein viewer on the InterPro website. Since then, we have enhanced the visibility of AntiFam by allowing users to browse AntiFam families directly and by creating individual pages for each family. This enhancement enables users to explore these families in the same way as other InterPro entries and member database signatures, including the ability to list all sequences in UniProtKB that are matched by a given family. Additionally, we regularly provide UniProt with a list of UniProtKB entries identified as spurious based on AntiFam detection.

### InterProScan improvements

Sequence search in InterPro is powered by the InterProScan software. InterProScan takes protein or nucleic acid sequences and searches them against InterPro's predictive models, which are provided by its member databases.

### Representative domains

As previously mentioned, the InterPro website now includes representative domains to provide a clear overview of the domain organisation within a sequence. These domains are automatically selected to maximise coverage while minimising overlaps between domains. InterProScan has been updated to report representative domains in both the JSON and XML output files. A new boolean ‘representative’ property or attribute has been introduced for each match, allowing users to filter and retain only the representative domains during downstream analyses.

### InterProScan docker image for cross-platform compatibility

InterProScan is only supported on Linux systems, but we aim to provide a software that runs out of the box on most personal devices and research institutes' high-performance computing (HPC) clusters. However, many researchers use macOS, which does not natively support InterProScan, and others may have systems that lack the necessary Linux libraries or have outdated versions of these dependencies.

To address these challenges, we created a Docker image of InterProScan, available on DockerHub (https://hub.docker.com/r/interpro/interproscan). Docker allows us to package InterProScan with all required dependencies in a container that can be easily deployed on any system that supports Docker, regardless of the underlying operating system. This ensures that researchers using macOS or systems without the appropriate Linux libraries can still run InterProScan without the need for complex installations or troubleshooting.

Furthermore, for those using containerisation tools like Singularity, SingularityCE, or Apptainer, the Docker image of InterProScan can be pulled directly from DockerHub and used seamlessly, just as it would be with Docker. This ensures that InterProScan is accessible across a wide range of computing environments, including HPC clusters where Singularity is commonly used. By providing a Docker image, we enable broader accessibility and more consistent performance for the research community.

### Extending PANTHER annotations

PANTHER has been integrated into InterProScan for over 15 years, traditionally using subfamily HMM scoring to assign sequences to specific subfamilies. However, starting with the release of InterPro 91.0 (October 2022), subfamily HMM scoring was replaced by TreeGrafter (50), a method that annotates protein sequences using pre-annotated phylogenetic trees. TreeGrafter not only outperforms subfamily HMM scoring in accurately assigning subfamily membership, but it also provides highly specific GO term annotations based on these reference phylogenetic trees. InterProScan now reports both the PANTHER family match and the tree graft location, along with subfamily annotations derived from the tree graft.

Starting with InterPro 95.0 (June 2023), GO terms associated with PANTHER tree nodes are reported in InterProScan, similar to how GO terms are associated with InterPro entries (InterPro2GO). These GO annotations are obtained from manually curated, explicit models of function evolution in protein families, from the Phylogenetic Annotation using Gene Ontology (PAN-GO) project (51). This enhances the functional annotation provided by InterProScan, offering researchers more precise annotation of protein function.

Moreover, TreeGrafter is significantly more computationally efficient than subfamily HMM scoring. It takes 13.8 hours to annotate the human proteome with InterProScan using PANTHER 15.0 and subfamily scoring, while it takes only 4.6 h with InterProScan using PANTHER 18.0 and TreeGrafter, demonstrating a useful improvement in efficiency.

### Monitoring and improving performances

The performance of InterProScan is largely dependent on the efficiency of the individual binaries used for each member database, the size of these databases (such as the number of profile HMMs), as well as the memory and CPU resources available on the system. When running InterProScan on a HPC system, performance is also influenced by variability in system performance due to factors such as system load, network latency or I/O contention.

Computing is a major contributor to energy consumption, and thus is an important source of carbon emissions. In the context of the global climate crisis, it is imperative that individuals and organisations find ways to assess and then reduce the carbon footprint of their work.

Running InterPro is a computationally intensive activity and, as such, has environmental impacts through carbon dioxide emissions. Between January 2023 and June 2024, we processed over 217 million sequences that were added to UniParc. The resulting annotations are made accessible to InterProScan via a lookup web service, which helps to avoid redundant and wasteful recalculations. When running InterProScan with the match lookup enabled (the default setting), the programme checks whether each input sequence has already been annotated and uses the pre-calculated annotations if available, instead of performing a local scan.

**Figure 6.**
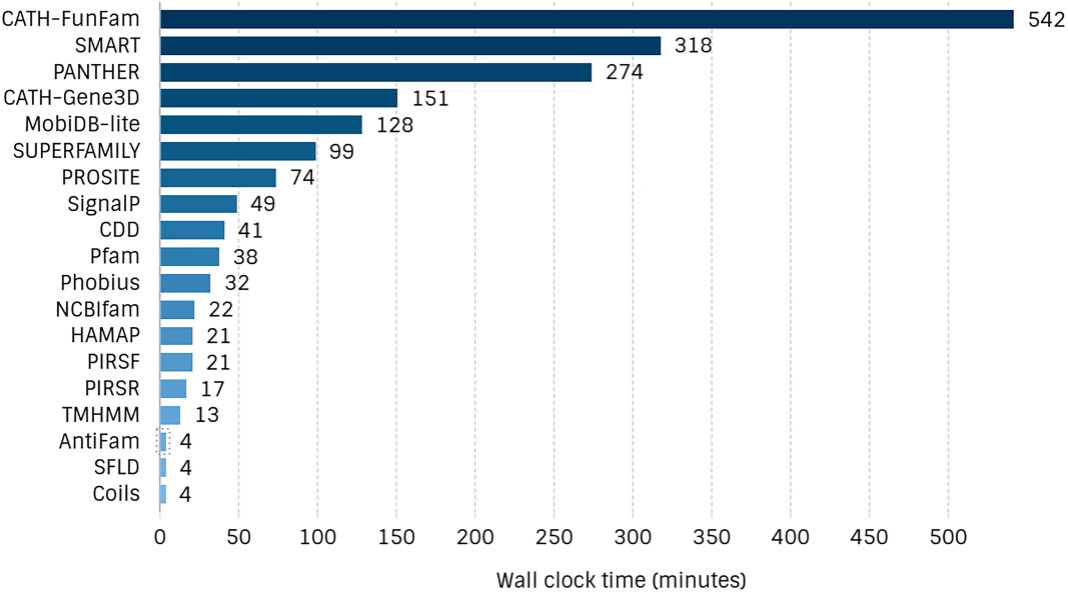
Wall clock time (minutes) consumed by individual member databases to annotate the human proteome using InterProScan with the match lookup disabled. Significant disparities in processing times are evident across the databases, highlighting the computational demands of specific analyses.

We continuously monitor the compute time of individual member databases within InterProScan to identify opportunities for optimisation and efficiency improvements (see Figure 6). Our goal is to minimise the environmental impact of our computational processes while maintaining the high standards of sequence analysis that our users depend on.

There is a significant variation in the wall clock time consumed by different member databases within InterProScan. MobiDB-lite, a resource focused on predicting intrinsic disorder in proteins, accounted for a compute time of two hours when annotating the human proteome with InterProScan with the match lookup disabled. Although significant, it is not the highest, with CATH-FunFam consuming the most compute time at nine hours. MobiDB-lite generates a consensus by integrating several predictors, such as DisEMBL (52), ESpritz (53) and IUPred (54). MobiDB-lite is implemented in Python 3 and performs system calls to execute the individual predictors, which are distributed as standalone scripts or binaries. This results in a nested and complex series of system calls, coupled with the generation of numerous temporary files, leading to suboptimal performance. Additionally, some predictors require Python 2, which has not been maintained since January 2020, necessitating the installation of both Python 2 and Python 3 on user systems.

To address these inefficiencies, we developed IDRPred (https://doi.org/10.5281/zenodo.13735975), a fast drop-in replacement for MobiDB-lite, and integrated it into InterProScan. The results are still labelled as MobiDB-lite, and the authors of MobiDB-lite have adopted this fork, which will become the official version. By replacing the original implementation with IDRPred, InterProScan annotates the full human proteome in 90 min instead of 128 min.

By focusing on optimising time-consuming processes, such as those in MobiDB-lite, we can significantly reduce the overall computational footprint of InterProScan, thus contributing to our efforts in lowering carbon emissions associated with large-scale sequence analysis.

### Extending protein annotations using deep learning

As part of a collaboration with Google Brain, we integrated ProtENN2 annotations in InterPro 91.0 (released in October 2022). ProtENN2, trained on Pfam data, uses convolutional neural networks to annotate protein sequences with Pfam family labels (55). By supplementing the traditional Pfam annotations generated by InterProScan with those provided by ProtENN2, we were able to increase the overall number of sequences that can be labelled with functional annotations. These ProtENN2 annotations, labelled as Pfam-N, were displayed in the InterPro protein viewer, and made accessible via the InterPro REST API and the EMBL-EBI FTP for download.

More recently, Google DeepMind developed a new transformer-based model, which has demonstrated a significant improvement in both prediction accuracy and coverage (Meng-Papaxanthos *et al.*, in preparation). This model was trained on InterPro 100.0 data, including Pfam 37.0. The resulting Pfam-N labels were made available in InterPro with the release of InterPro 101.0 in July 2024.

As with the previous version of Pfam-N, these annotations are now readily accessible through multiple platforms. Users interested in visually exploring protein domains can view the annotations in the InterPro protein viewer. For those who wish to integrate these annotations into their own websites, tools, or downstream analyses, the InterPro REST API provides access. Additionally, users who require annotations for all sequences in UniProtKB can obtain them via bulk download from our FTP site. These multiple access points ensure that the enhanced Pfam-N annotations are available to a broad range of users, from researchers conducting large-scale data analysis to developers building bioinformatics applications.

**Figure 7.**
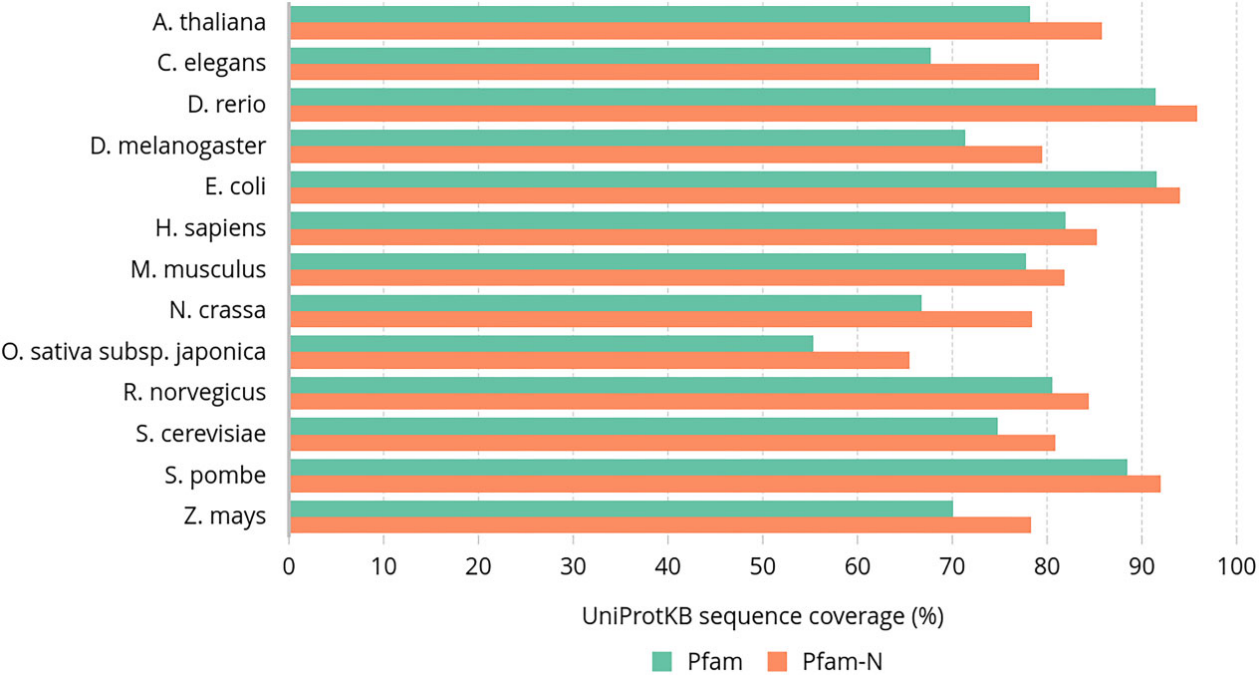
Comparison of annotation coverage between Pfam and Pfam-N across key species and model organisms. For each species, Pfam-N consistently shows higher annotation coverage compared to Pfam, highlighting its enhanced ability to annotate a larger proportion of protein sequences, thereby providing more comprehensive functional information across these organisms.

Pfam-N offers a more comprehensive coverage of UniProtKB sequences compared to Pfam, providing at least one annotation for 85% of sequences in UniProtKB 2024_04, representing an 8% increase over Pfam. It also annotates more sequences than Pfam across several key species and model organisms (see Figure 7). Notably, Pfam-N annotates 12 000 proteins from Swiss-Prot, the reviewed section of UniProtKB, that Pfam does not annotate. Additionally, Pfam-N provides annotations for over 11 million sequences that are not annotated by Pfam or any other InterPro member database, thereby enhancing the comprehensive annotation of known protein sequences.

## Conclusion

InterPro continues to evolve as a vital resource for protein sequence classification and functional annotation. The database has made significant progress in integrating new data sources, enhancing analytical capabilities and improving user accessibility.

By bridging the gap between raw sequence data and functional insights, InterPro and InterProScan play a crucial role in advancing our understanding of the protein universe, thereby supporting a wide range of biological research activities.

The field of protein classification has been transformed by the rise of powerful protein structure prediction methods like RosettaFold and AlphaFold. These advances have dramatically improved our ability to predict protein structures, which, in turn, has been instrumental in the creation of Pfam families and families in other databases. These families are subsequently integrated into InterPro entries, enhancing the comprehensiveness and accuracy of the database.

The InterPro REST API remains a popular tool for programmatic access to InterPro data, facilitating its integration into third-party applications and pipelines. However, we have observed an increase in the number of users submitting tens of thousands of requests in a very short period, which can cause the API to become temporarily unavailable for all users. In an era where more public resources are being utilised for AI training, we encourage users to download data in bulk from our FTP site or to start querying our API gradually, then ramp up their usage.

As the volume of sequence data continues to grow rapidly, we have managed to maintain coverage of UniProtKB and UniParc, although we are lagging in expanding this coverage. While achieving 100% sequence or residue coverage is neither a realistic nor sensible goal, we recognise the need to address this challenge. Although the speed of sequence annotation using profile-HMM scoring and other traditional methods is important, it is not currently the main bottleneck in creating InterPro entries and integrating member database signatures. The most significant limiting factor remains the manual curation steps. We have begun using LLMs to annotate member database signatures that lack descriptions, which are then automatically integrated into new InterPro entries. However, this process is dependent on the performance of these LLMs, and the best-performing models are often commercial, limiting our capacity to massively describe protein families and update the generated descriptions.

Ultimately, InterPro relies on the underlying member database signatures it integrates. The coverage of sequence databases by InterPro is, at best, the union of the coverage of its individual member databases. While AI has shown promise in improving our ability to identify homologous in existing protein families, exemplified by Pfam-N and its integration in InterPro, the field is rapidly evolving, and no single model or deep learning architecture has yet emerged as the clear choice for standardisation. Furthermore, while we currently have these annotations available, it is essential that these models be made publicly available and integrated into InterProScan so that end-users can benefit from them.

These are exciting times for the scientific community, and we anticipate significant strides in the coming years towards a more complete and accurate classification of protein families and domains.

## Data Availability

InterPro releases are published every eight weeks. All data are freely available from the main website (https://www.ebi.ac.uk/interpro), in bulk from the FTP site (https://ftp.ebi.ac.uk/pub/databases/interpro/), and programmatically via the REST API (https://www.ebi.ac.uk/interpro/api/). The latest version of InterProScan can be downloaded from the InterPro website (https://www.ebi.ac.uk/interpro/download/interproscan/). Previous versions are available on the FTP site (https://ftp.ebi.ac.uk/pub/software/unix/iprscan/5/). InterProScan is distributed under the open source Apache 2.0 licence.
